# Impact of finger biophysical properties on touch gestures and tactile perception: Aging and gender effects

**DOI:** 10.1038/s41598-018-30677-2

**Published:** 2018-08-22

**Authors:** A. Abdouni, R. Vargiolu, H. Zahouani

**Affiliations:** Univ Lyon, Laboratoire de Tribologie et Dynamique des Systèmes-LTDS UMR-CNRS 5513, ECL-ENISE, F-69134 Ecully, France

## Abstract

The human finger plays an extremely important role in tactile perception, but little is known about the role of its biophysical properties (mechanical properties, contact properties and surface topography) in tactile perception. In addition, the touch gestures used to perceive an object’s properties differ among people. We combined studies on the biophysical properties and the vibrations measured from the human finger to understand the age and gender effects on the tactile perception and the difference between the touch gestures. In addition, a new algorithm, Mel-frequency cepstral coefficients (MFCCs), was used to analyze the vibratory signal obtained from the physical contact of the finger, and a surface is proposed and validated. The values obtained regarding the correlation between the tribohaptic system results and the biophysical properties show that the Young’s modulus and the surface topography are the most important. An inverse correlation was observed between the MFCC and the tactile perception. This last observation explained the results of better tactile perception with left to right touch gestures. It also demonstrated a better tactile perception for women.

## Introduction

People can perceive the properties, contact, shape, hardness and roughness of an object within a few seconds of physical contact. They use their experience of objects and knowledge of similar aspects to describe what they perceive. The nature of how people explore and come into contact with objects is essential to their perceptions of the object. To determine different object properties, people use specific gestures called ‘exploratory procedures’^1^. To perceive texture, hardness, temperature, weight, global shape, volume and exact shape, people typically choose what they think is the most effective exploratory procedure. For example, to perceive the roughness of an object’s surface, people spontaneously rub the object. Different directions can be chosen to rub the object, such as lateral, longitudinal, and circular directions. In this study, we focused on the surface perception of objects and the gestures used by the participants as a function of age and gender.

Human touch involves physical contact between the fingers and objects, so it is dependent on tactile skin and its biophysical properties. When a finger slides across an object, vibrations are generated that propagate through the skin, thus activating mechanoreceptors embedded at various depths^2–6^. Four types of mechanoreceptors, Meissner’s corpuscles (RA), Pacinian corpuscles (PC), Merkel’s disks (SA I) and Ruffini’s corpuscles (SA II)^7,8^, are responsible for touch sensation, pressure, vibrations and cutaneous tension that result from mechanical deformations due to contact between the finger and the object touched. This deformation is converted into different specific electrical signals^9,10^. Surface texture is composed of three main parameters: roughness, compliance and viscoelasticity^11,12^. When a finger moves over the surface of an object, it perceives surface texture properties through vibrations generated in the skin^13–16^. It has been observed that Ruffini’s corpuscles are very sensitive to stretching^17^, whereas Merkel’s disks detect small pressure^18^. The roughness and vibration sensing relation has been investigated^19^, and Pacinian corpuscles and Meissner’s corpuscles are presumed to detect high and low frequency vibrations, respectively. However, Ruffini’s corpuscles and Merkel’s disks could not be excluded from roughness measurements^17^. In the literature, certain studies have addressed the weakening of human touch with age and differences due to gender. As we get older, our sense of touch diminishes for many reasons. First, a reduction in central nervous system activation has been investigated^20,21^. Second, a significant decrease in the density of Meissner’s corpuscles has been illustrated^22,23^. Third, the vibrotactile detection threshold (VDT), which is the minimum detectable level of vibration, increases as a function of age^24^.

Many techniques have been proposed to study the effect of surface texture on tactile perception. In the literature, many researchers have addressed the dynamic friction coefficient, µ, by measuring the normal and tangential forces between a finger and an object^25–28^. Other studies have used very sensitive sensors to investigate the vibration signals generated from a finger during the touch process^27,29^. The vibratory level, *L*_*a*_, and the dynamic friction coefficient, µ, are the parameters most commonly used to study surface texture by analyzing the vibratory signals and force signals, respectively.

In our previous study, we investigated aging and gender influences on the biophysical properties of the human finger^30^. The results obtained showed significant differences in finger mechanical properties, contact properties and surface topography. In addition, the results demonstrated a clear anisotropy of mechanical properties. In this study, we used a haptic tribometer system to address the role of the anisotropy of finger mechanical properties on tactile perception. In addition, the effects of age and gender on tactile perception are studied using the biophysical properties of the human finger. Finally, a new algorithm for analyzing the vibratory signal obtained from the contact between a finger and a surface is proposed and validated.

## Materials and Methods

A panel of forty French volunteers (20 women and 20 men) participated in the experiments. All the subjects were white-collar workers, and all the measurements were performed *in vivo* and were noninvasive. For efficient analysis, we used the same dataset of participants as a previous study^30^, which means that we already had the biophysics properties of their fingers. The database was divided into four age groups (26 ± 3, 35 ± 3, 45 ± 2, and 58 ± 6 years old) of five persons each. All volunteers were trained to control the normal force applied and the speed at which they slid their finger on a surface. They were adequately informed of the aims, methods used and potential risks of the study, and they gave their written informed consent to the protocol.

**Figure 1:**
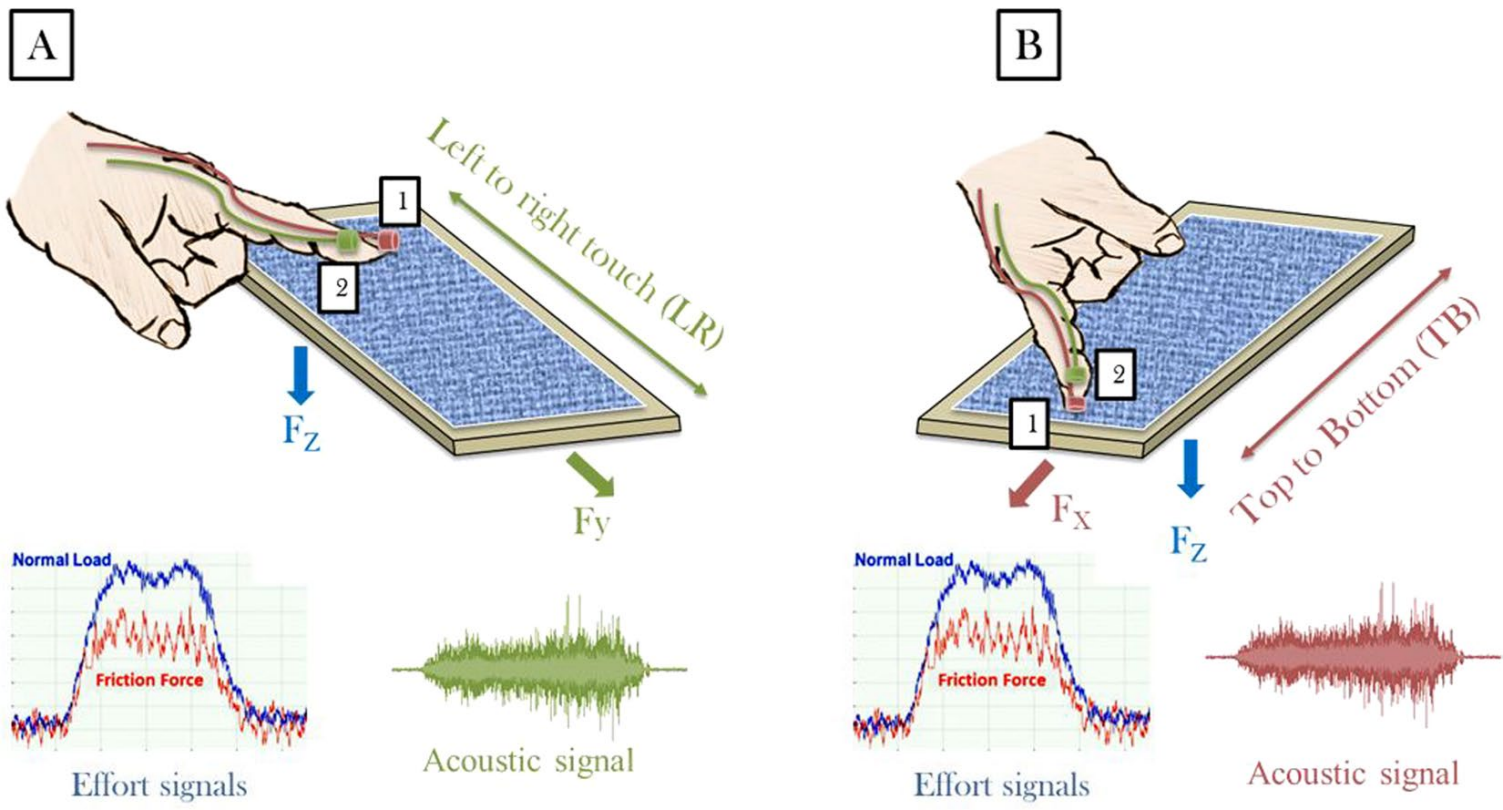
Touch gesture with haptic device. The accelerometer detecting the vibrations was glued each time to the volunteer’s finger. The accelerometer had a single axis, and it was parallel to the contact plane during the test. Normal and tangential forces were measured by two stress gauge sensors placed just below the support of the surface to be tested. (**A**) Left to right touch gesture (the red accelerometer). (**B**) Top to bottom touch gesture (the green accelerometer). (1) Accelerometer for top to bottom touch gesture and (2) accelerometer for left to right touch gesture.

A haptic tribometer system developed, patented and published previously by our team was used to characterize the vibrations transmitted by the finger during a tactile perception test^29,31–33^. In this study, two accelerometers were glued each time to the volunteer’s finger, and the range of vibrational frequencies was highly consistent with the mechanoreceptor frequency band (1–500 Hz) (see Fig. 1). To avoid causing minimal disturbance of the measurements, the accelerometer is preferably bonded to the friction finger, including for example, using a cyanoacrylate glue. This glue has the advantage of little or no mitigating or disturbing effects on the measurement of vibrations and is harmless to human skin. The accelerometer used to sense the vibratory signal at the finger pad was attached to a side of the finger opposite to the direction of movement of the finger on the surface. Such a position of the accelerometer has the advantage of capturing the tangential vibrations of the whole volume at the outlet of the pulp during the translation thereof. The proposed system ensured physical contact between the human finger and two accelerometers during the friction test. The accelerometers had the following characteristics: a mass of 0.14 g, diameter of 3.58 mm, gain sensor of 1 V/m/s² and vibration sensor sensitivity of 0.5 pC/m/s^2^. Each accelerometer had a single axis and was parallel to the contact plane during the test. Two accelerometers were used to perform lateral and longitudinal axis measurements. For each touch gesture, only one accelerometer was activated (see Fig. 1). This equipment included two stress gauge sensors that allowed simultaneous measurements of the normal and the tangential forces. The maximum effort was 0.8 N with a resolution of 1 mN.

In the literature, the friction coefficient, power spectral density (PSD) and average vibratory level (La(dB)) are the quantitative parameters most often used^29,32–35^. PSD is a parameter related to the spatial resolution of the human finger, which is identified as the characteristic wavelength corresponding to the maximum power spectral density. For a constant normal force, the vibratory level, *L*_*a*_, allowed us to compare the vibration received by the finger as a function of the nature of the surface touched. The average friction coefficient *µ* obtained from 10 cycles was calculated using the ratio between the friction force, *F*_*x*_, and the normal force, *F*_*N*_, as follows:

$$\mu =\frac{{F}_{x}}{{F}_{N}}.$$

In addition, in order to provide a quantitative value to the vibrations, the average acoustic vibratory level was defined as follows:

$${L}_{a}=20\,\mathrm{log}(\frac{{A}_{RMS}}{{A}_{ref}}),$$

where *L*_*a*_ is the acoustic vibratory level, *A*_*RMS*_ is the root mean square value of the acceleration in m/s², and *A*_*ref*_ is the smallest acceleration that can be detected by the sensor, which is 10^−6^ m/s².

**Figure 2:**
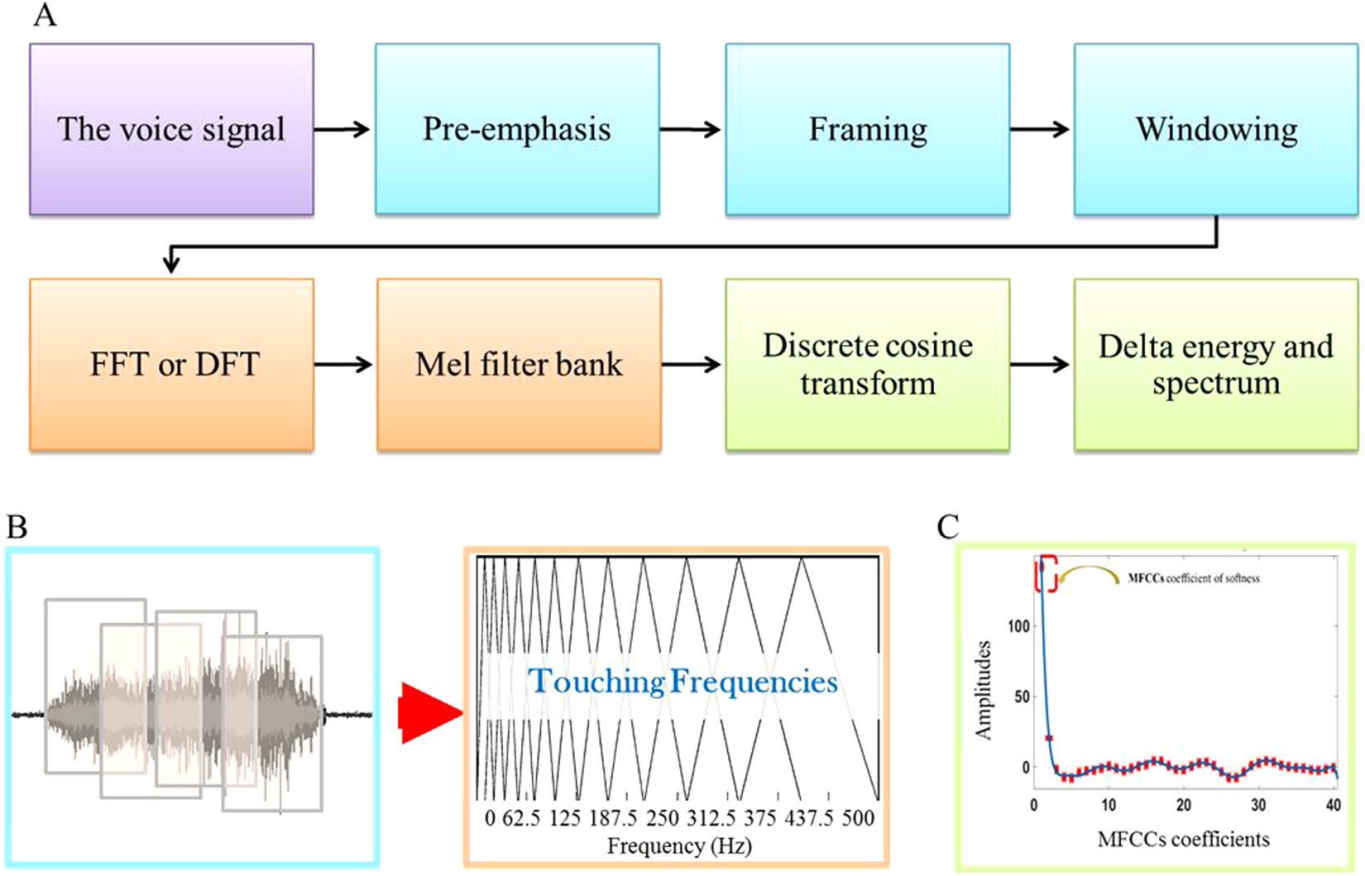
Mel Frequency Cepstral Coefficients (MFCCs) algorithm. (**A**) Block diagram of the MFCC algorithm. Four phases can be identified. 1- Read the vibratory signal sample. 2- Split the audio signal into distinct “frames”. 3-Compute the Mel-spaced filterbank; this is a triangular filter (illustration in Figure B) that we apply to the periodogram power spectral estimate from step 2. 4-Take the Discrete Cosine Transform (DCT) of the log filter bank energies to give cepstral coefficients (illustration in Figure C). (**B**) A Mel-filterbank adapted to correspond to the mechanoreceptors frequencies. (**C**) Cepstral coefficients of a vibratory signal. The highlighted coefficient is the sum of how much energy exists in the range of (0 to 500 Hz) and is indicated in the softness coefficient.

*L*_*a*_ and µ were calculated in this study. The PSD results were very similar to those for *L*_*a*_. Consequently, we decided to keep only *L*_*a*_ to analyze the vibratory signal. In addition, a new algorithm was proposed that uses Mel-frequency cepstral coefficients (MFCCs) to analyze the vibratory signal. The MFCC algorithm is the most commonly used feature extraction method in automatic speech recognition^36^. In addition, MFCCs have been used in other domains, such as palm print, music modeling and music instrument identification^37–40^. When using MFCCs in speech analysis, it has been determined that 8–14 coefficients are sufficient, and very often, 12 are chosen^41^. In the method proposed here, we tried to determine how many of these coefficients were necessary and useful for surface characterization. The accelerometer signal obtained during the touch process was a vibratory signal that resembles a sound signal. The MFCCs analyzed the vibratory signal according to the following steps^42–44^ (see Fig. 2A).

A vibratory signal changes constantly, so for the sake of simplification, this signal was assumed to be statistically stationary over short time scales, which is why we framed the signal into 20–40 ms frames. The frame duration was chosen to provide enough samples to obtain a reliable spectral estimation. The power spectrum of each frame was calculated using a periodogram. The latter played the role of the human cochlea (an organ in the ear), which vibrates at different points depending on the frequency of incoming sounds. The presence of different frequencies was identified by the location in the cochlea that vibrated. The cochlea has one limitation: it cannot discern the difference between two closely spaced frequencies. This effect becomes more pronounced as frequency increases. Therefore, the sum of all clumps of periodogram bins was taken. This sum indicated how much energy existed in various frequency regions. This summation in various frequency regions was performed by the Mel filterbank (see Fig. 2B). The first filter was very narrow and provided an indication of how much energy existed at low frequencies (0–500 Hz). As frequencies became higher, our filters widened since we became less concerned with variations. Once we obtained the filterbank energies, we derived their logarithm, which allowed us to use cepstral mean subtraction, a channel normalization technique (incorporating this scale made our features match more closely with human hearing), which uses the following equation:

$$M(f)=1125\,\mathrm{ln}\,(1+f/700).$$

The final step was to compute the discrete cosine transform (DCT) of the log filterbank energies. This step was performed because the filterbanks all overlapped, and the filterbank energies were notably correlated with each other. The DCT decorrelates the energies so that diagonal covariance matrices can be used to model the features. In this study, we focused on approximately how much energy occurred in small frequencies and at each point. The first coefficient is kept in this study, which is different than what is recommended in speech recognition (8–14 coefficients are sufficient)^43^ (see Fig. 2C). Finally, the first coefficient, the sum of all energies at low frequencies (0 to 500 Hz), showed the best correlation with the softness results, such as *L*_*a*_. This range of frequencies corresponded to the firing band of all mechanoreceptors, which means that the information obtained with MFCCs could be correlated to all types of stimuli. In this study, all the results presented with MFCCs used the first coefficient.

MatLab software was used for statistical data analysis. Analysis of variance (ANOVA) is a statistical model used to analyze the differences between and among groups and to determine whether the differences between the means are statistically significant. The data are considered statistically significant if p is less than the significance level defined (0.05 in our case). In our study, we used ANOVA to determine whether we had statistically significant differences as a function of age and gender, and significant differences are denoted with a ‘*’.

In statistics, the Pearson correlation coefficient (r) is a measure of the linear dependence (correlation) between variables. The Pearson correlation coefficient is used to determine the strength of a relationship between variables. The correlation is considered strong for an r value higher than 0.8. In this study, we used the r coefficient to determine whether we had a statistically linear correlation between age groups^45^. A correlation coefficient higher than 0.8 indicates a strong positive correlation (+), whereas a correlation lower than −0.8 is considered a strong negative correlation (−). ‘0’ indicates no linear correlation between the data (−0.8 < r < 0.8).

## Results and Discussion

### Validation of MFCC algorithm (handfeel panel/human finger)

Tissues and plastic samples were used to validate the MFCC algorithm. For both samples, we compared the results obtained with MFCCs and *L*_*a*_ with handfeel. Then, we used statistical analysis to evaluate and compare the performance of each algorithm.

#### Tissue samples

**Figure 3:**
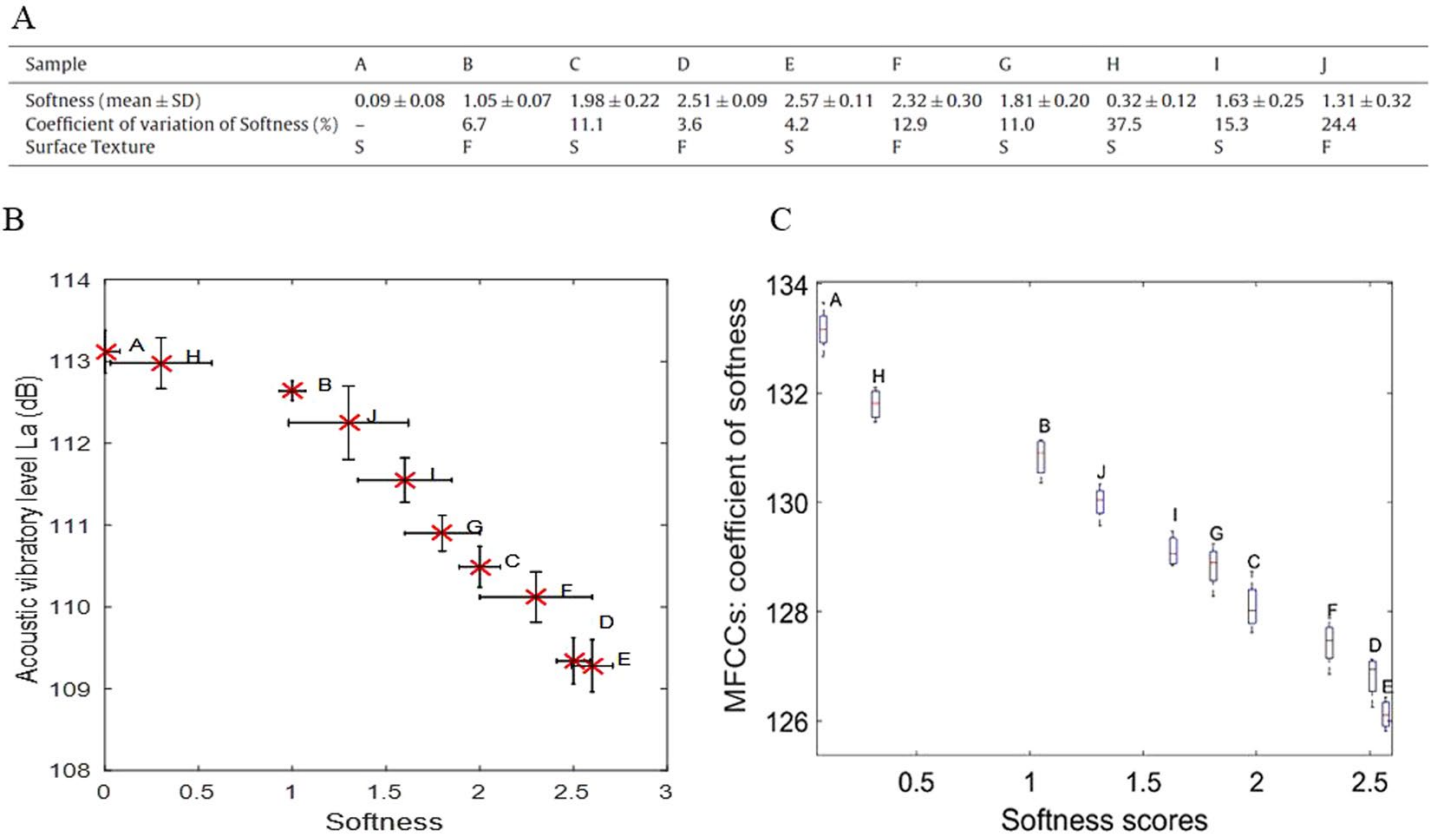
Comparison between the results obtained from the handfeel panel and those measured with the artificial finger: (**A**) The results of the handfeel panel relating to the surface softness and texture of ten bathroom tissues. For the surface texture, F stands for a fluffy tissue and S for a smooth one. As the volunteers had only two choices, they all agreed on the velvetiness (fluffiness) or the smoothness of each sample surface. The values of the softness estimated for each tissue sample corresponded to the mean ± standard deviation values obtained for each volunteer. (**B**) Acoustic vibratory level as a function of softness. (**C**) MFCCs as a function of softness. Figures (**A**) and (**B**) are taken from another study^33^. The results in Figure (**C**) are calculated based on the data of Figure (**B**). Figure (**C**) shows new results with MFCC. ANOVA and Pearson’s statistical analyses: (+for r > 0.8, - for r < −0.8) highly correlated, (0) for no correlation, *p < 0.05 for statistically significant. The statistics analysis shows better results for MFCC (r ≈ −1, p < 0.05) than the Vibratory level (r = −0.96, p < 0.05).

For validation of the MFCC algorithm, we used the handfeel sensory panel (trained panel) and acoustic level results from a previous study by our team (see Fig. 3)^33^. The new part in this article is that we used the MFCC algorithm to analyze the data collected previously (see Fig. 3A), and we compared these results to those obtained in the previous article with the vibratory level (see Fig. 3B).

In the previous study^33^, the handfeel panel volunteers were asked to rank the ten tissue samples as a function of their softness. To evaluate the softness of the bathroom tissues, the panel volunteers had to assign a score for the ten tissues samples. To rank the ten tissue samples, the handfeel panel had at their disposal four reference fabric samples. Each reference sample corresponded to a score: 0, 1, 2, or 2.5, where 0 corresponded to the least soft tissue and 2.5 corresponded to the softest tissue^33^.

The evolution of the acoustic vibratory levels of the ten paper samples as a function of their softness evaluated by the handfeel panel is reported in Fig. 3B. The results indicate that softness was highly negatively correlated with the acoustic vibratory level with a confidence level of 95% (R = −0.96, p < 0.05). For a softness feeling estimated by the handfeel panel from 0 to 2.6, the acoustic vibratory level values measured decreased from 113.2 dB to 109.2 dB. The results are in good agreement with those of Zahouani *et al*., who showed that the sound level of the skin during friction was a good criterion for assessing skin softness^46^. Figure 3C shows the new results obtained with MFCCs, which are perfectly negatively correlated with the handfeel panel with a confidence level of 95% (R ≈ −1, p < 0.05). The MFCCs decreased from 134 to 125. These results showed the high performance of MFCCs in describing softness as it presented better correlation and a larger difference between the minimum and the maximum values of MFCC. This last result means that MFCC offers better classification than the vibratory level. Indeed, we can conclude that the softest sample corresponds to the minimum vibratory parameter (MFCCs and *L*_*a*_).

#### Plastic samples

**Figure 4:**
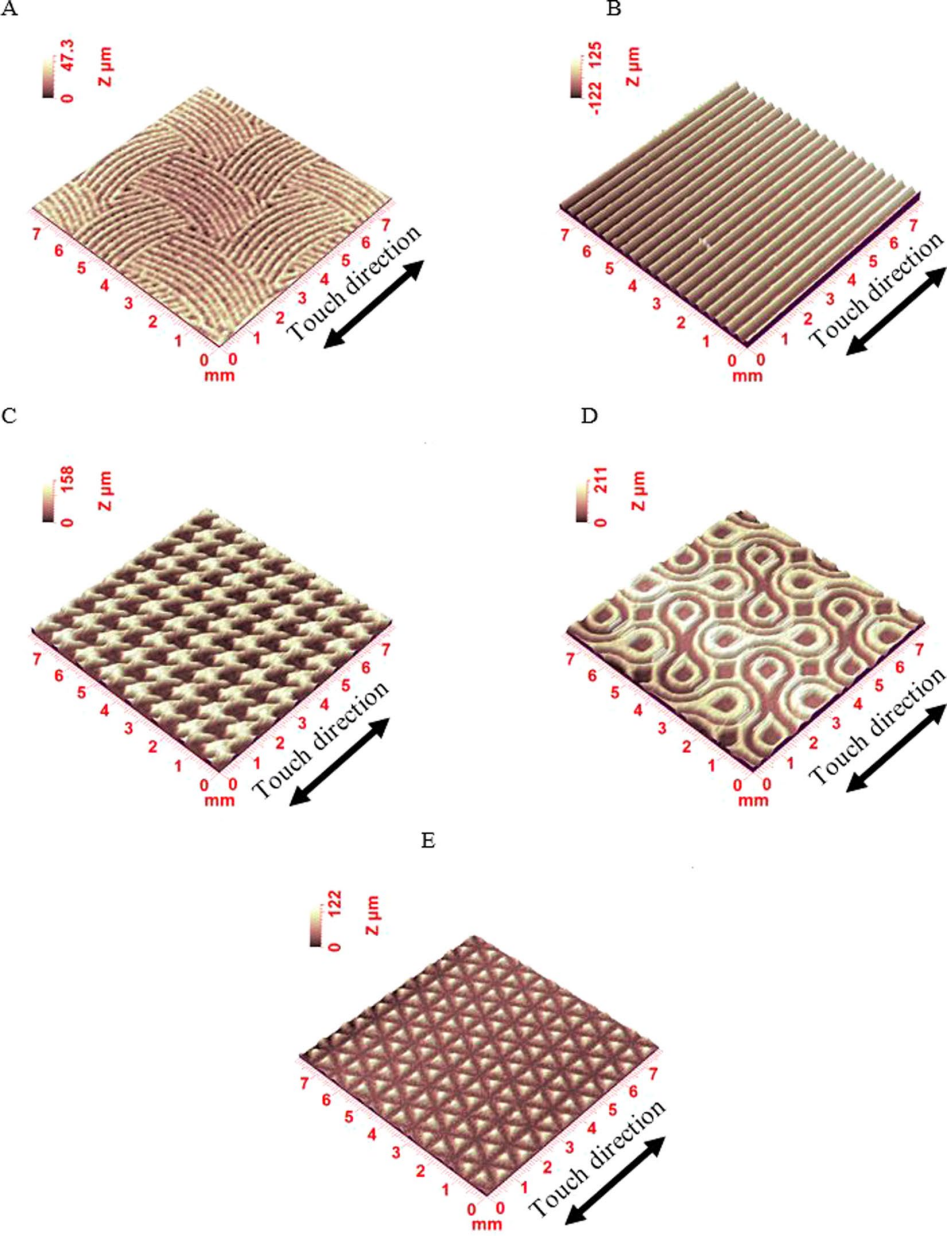
Confocal scans of five plastic sample topographies. The five plastic surfaces had the same stiffness but different textures with different tactile properties. The support material was PVC, and the printed material was UV light ink. The digital printer was the Mímaki brand with a resolution of 1,200 bpi.

We also validated the MFCC algorithm on plastic samples. All volunteers (described previously in section 2) were monitored under the same experimental procedure for approximately 40 min. This procedure was organized chronologically as follows. First, the subject had to stay undisturbed for 15 min in a room at a temperature of 23 ± 1 °C with 55% relative humidity (RH) before cleaning his/her right forefinger with a piece of tissue. Then, the touch experiments were conducted in the same room. The experimental conditions used with the human finger are summarized as follows: 10 uniform forward translations in the lateral direction and then in the longitudinal direction, normal force: 0.3–0.4 N, sliding speed: 20–30 mm.s^−1^, and friction length: 20 mm. These conditions are frequently observed in the literature and correspond to classical human handling conditions^47,48^. These healthy volunteers were asked to evaluate the tactile properties of the tissues based on a softness descriptive criterion. In this study, we focused on the softness feeling of plastic surfaces. Softness is one of the most important parameters for judging sensory quality. The softness was defined on five levels of a haptic scale according to softness. These levels were quantified by scores 1, 2, 3, 4 and 5 in order of softest to least soft. The five plastic surfaces had the same stiffness but different textures with different tactile properties (see Fig. 4). The plastics were made by Digitex technology from Eschmann Textures. The support material was Polyvinyl chloride (PVC), and the printed material was UV light ink. The digital printer was the Mímaki brand with a resolution of 1,200 bpi. The samples were placed in random order. The tests were performed with eyes blindfolded. The panel graded the samples in two defined directions (left to right (LR) and top to bottom (TB)) as a function of their softness. The results are presented in Table 1. Plastic surfaces were used due to the possibility of having a very large variety of characteristics (hardness, topography, and surface energy). In addition, plastics are usually used to coat different products, which is why the most commonly touched surfaces in daily life are made of plastics, and comprehension of the evaluation of plastic quality is a pronounced task.

**Table 1:** Results of the panel related to surface softness classification of different plastic surfaces for two different touch gestures.

Touch gesture	Gender	Age groups	Sample softness (the mean ± SD)					Statistics (r, p)			
			A	B	C	D	E	La		MFCCs	
								r	p	r	p
Top to bottom touch	Men	G1	1.6 ± 0.3	1.3 ± 0.3	2.9 ± 0.3	4.1 ± 0.2	5.0 ± 0.0	0	*	+	*
		G2	2.1 ± 0.5	1.2 ± 0.2	2.4 ± 0.2	3.8 ± 0.3	5.0 ± 0.0	+	*	+	*
		G3	2.2 ± 0.3	1.5 ± 0.3	3.1 ± 0.4	3.9 ± 0.3	5.0 ± 0.0	0	*	+	*
		G4	1.2 ± 0.2	2.2 ± 0.3	4.2 ± 0.3	4.1 ± 0.2	5.0 ± 0.0	0	*	+	*
	Women	G1	1.6 ± 0.3	1.3 ± 0.3	3.6 ± 0.6	2.3 ± 0.3	5.0 ± 0.0	+	*	+	*
		G2	1.2 ± 0.2	2.5 ± 0.3	4.2 ± 0.3	3.1 ± 0.1	5.0 ± 0.0	+	*	+	*
		G3	1.8 ± 0.3	1.3 ± 0.3	3.5 ± 0.1	3.2 ± 0.2	5.0 ± 0.0	0	*	+	*
		G4	1.4 ± 0.3	2.1 ± 0.3	3.2 ± 0.2	4.1 ± 0.3	5.0 ± 0.0	0	*	+	*
Left to right touch	Men	G1	1.1 ± 0.1	2.0 ± 0.5	3.8 ± 0.3	3.1 ± 0.1	5.0 ± 0.0	+	*	+	*
		G2	1.2 ± 0.2	2.2 ± 0.2	3.9 ± 0.3	3.2 ± 0.2	5.0 ± 0.0	+	*	+	*
		G3	1.3 ± 0.3	2.1 ± 0.1	4.1 ± 0.3	3.0 ± 0.3	5.0 ± 0.0	0	*	+	*
		G4	1.2 ± 0.2	1.8 ± 0.3	3.9 ± 0.3	2.9 ± 0.2	5.0 ± 0.0	0	*	+	*
	Women	G1	1.0 ± 0.0	2.2 ± 0.3	4.2 ± 0.3	3.1 ± 0.4	5.0 ± 0.0	+	*	+	*
		G2	1.2 ± 0.2	2.3 ± 0.3	3.6 ± 0.6	2.9 ± 0.3	5.0 ± 0.0	0	*	+	*
		G3	1.3 ± 0.3	2.2 ± 0.2	3.5 ± 0.1	2.8 ± 0.2	5.0 ± 0.0	+	*	+	*
		G4	1.2 ± 0.2	1.9 ± 0.2	4.1 ± 0.2	3.2 ± 0.2	5.0 ± 0.0	0	*	+	*

The samples’ softness scores were quantified by 1, 2, 3, 4 and 5 in order of softest to least soft. Those scores correspond to the mean value for each age group ± the standard deviation. ANOVA and Pearson’s statistical analyses: (+for r > 0.8, – for r < −0.8) highly correlated, (0) for no correlation, *p < 0.05 for statistically significant. The four age groups (G1, G2, G3, and G4) correspond to (26 ± 3, 35 ± 3, 45 ± 2, and 58 ± 6 years old), respectively.

For a softness feeling estimated by the handfeel panel from 1 to 5 (from the softest to the least soft, see Materials and Methods section), the measured acoustic vibratory level values increased from 106 dB to 124 dB, and the measured MFCCs values increased from 122 to 148. The results are in agreement with the previous section, where the softest sample corresponded to the minimum vibratory parameter. As in part (a), a higher correlation between MFCCs and the softness feeling was obtained compared to the correlation between *L*_*a*_ and the softness panel scores, as shown in Table 1.

The acoustic level *L*_*a*_ is known as a parameter capable of describing the softness of a surface by analyzing the vibratory signal obtained for a human finger. The MFCC algorithm is proposed to address the same problem as *L*_*a*_. The statistical analysis showed the performance of *L*_*a*_ and MFCCs as a function of the handfeel panel. The results given in Table 1 and Fig. 3 show perfect correlations between MFCCs and the panel results (|r| > 0.8, p < 0.05) for tissue and plastic samples. These findings confirm the high performance of the MFCC algorithm compared to *L*_*a*_ in obtaining representative values of softness from vibratory signals. Therefore, for the rest of this study, we kept MFCCs and µ but gave priority to MFCCs due to their better performance.

### Vibratory parameters vs touch gestures (LR and TB) and the biophysical properties of the human finger: Age and gender effects

**Figure 5:**
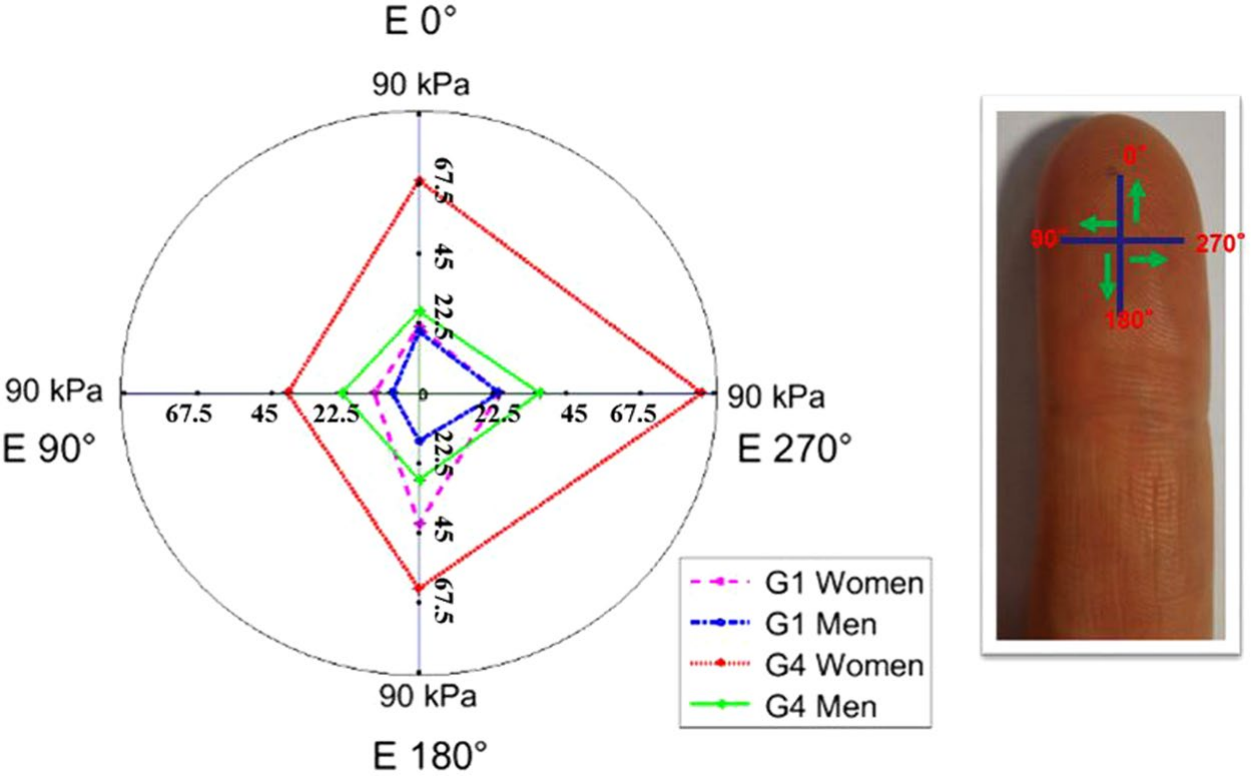
Illustration of the anisotropy of the finger’s mechanical properties for both men and women of the youngest and the older groups^30^. The two age groups (G1, G4) correspond to (26 ± 3 and 58 ± 6 years old), respectively, for men and women (this illustration is taken from our previous study^30^). The evolution of Young’s modulus with age is anisotropic as a function of the direction measured. The results demonstrate a higher E for the exterior part of the finger skin (0°, 270°). The exterior part of the finger is more exposed to the environment and to repeated friction in daily life (i.e., writing); therefore, increasing anisotropy of mechanical properties with age can be observed^30^.

Pronounced differences in the human finger’s biophysical properties between men and women were encountered in the previous study^30^. All the results in this paragraph have been obtained in our previous article^30^. The mechanical properties (elastic modulus), contact properties (adhesive force) and arithmetic mean of the surface topography (multiscale arithmetic mean of roughness amplitude *(SMa)*) measured in 40 volunteers were investigated. All results showed significant differences between men and women and as a function of age. Regarding the results of contact characteristics (i.e., adhesive force), the values obtained were significantly higher for women than for men. For the mechanical properties (i.e., Young’s modulus *E*), a significant and positive correlation with age was observed and found to be higher for women than men. In addition, the evolution of Young’s modulus with age was anisotropic as a function of the direction measured (see Fig. 5). The results demonstrate a higher Young’s modulus for the exterior part of the finger skin. For topography analysis, a different age effect was presented in the comparison between men and women. In this study, the gender effect on tactile perception was investigated on the basis of the differences in these biophysical properties.

To explain the results obtained, we reviewed the literature. Concerning the vibratory parameters (MFCCs and *L*_*a*_) and Young’s modulus relationship, a positive correlation was demonstrated between *L*_*a*_ and Young’s modulus. In 2010, Ben-abdelounis^49^ demonstrated that the acoustic level is directly proportional to Young’s modulus.

In 1973, Takahashi^50^ showed that the relation between sound pressure, *Lp(dB)*, and surface roughness, *Ra*, is a linear and increasing function according to a logarithmic law of the form: $Lp\approx 20\,{\mathrm{log}}_{10}R{a}^{n}$ for a cylinder/plane contact. This result shows that sound pressure is directly proportional to surface roughness, i.e., $La\propto Ra.$

The results are described in the following sections, which successively address the effect of biophysical properties (contact properties, mechanical properties and topographic characterization) on the tactile perception for each touch gesture as a function of age and gender. In this part, one plastic surface is used (sample D in Fig. 4).

#### Left to right touch gesture vs biophysical properties: Age and gender effects

**Figure 6:**
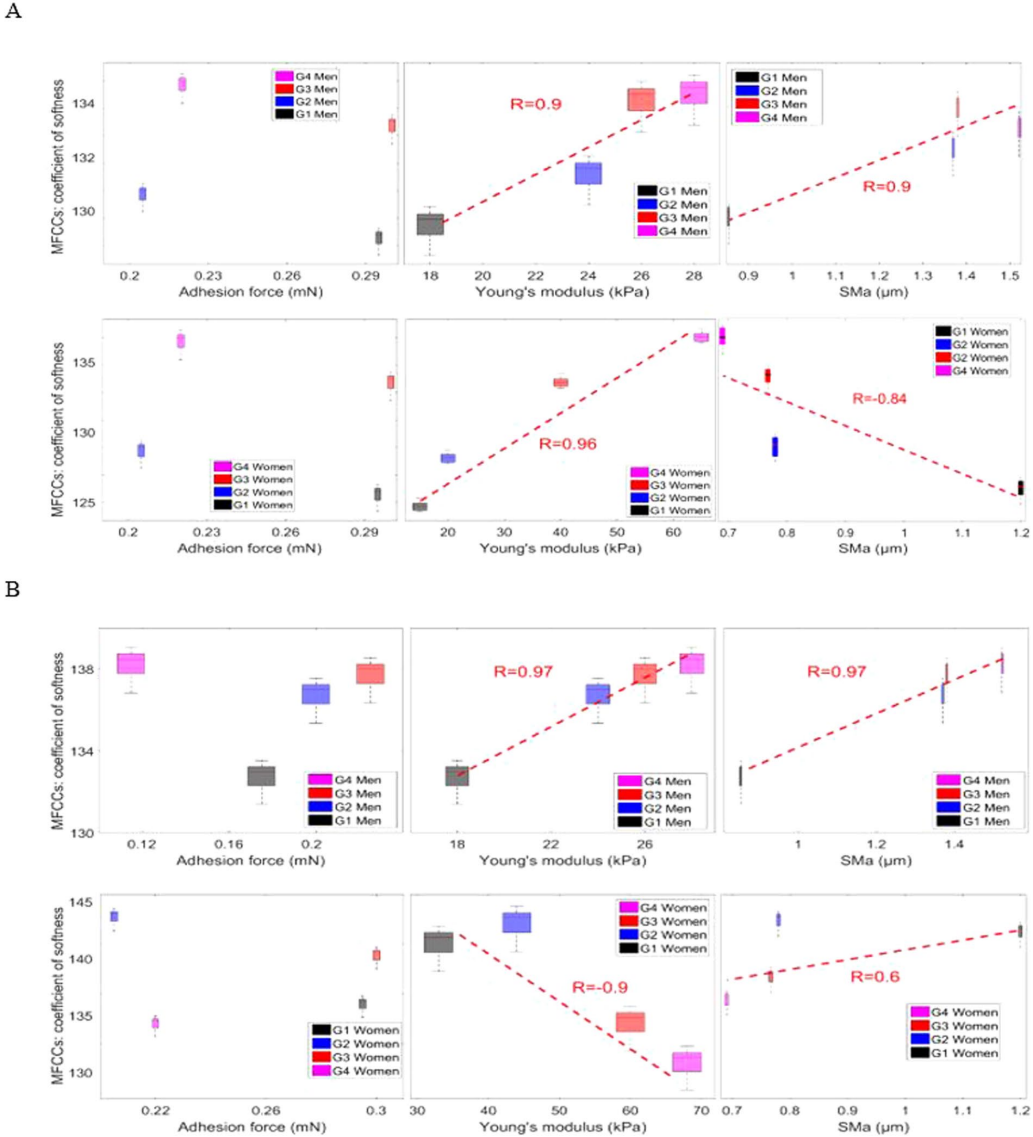
Correlation results between haptic touch parameter (MFCCs) and biophysical properties (adhesion force, Young’s modulus and topographic characterization SMa) for the human finger as a function of age are illustrated. The results are obtained with sample D. The adhesive force, Fad, is the force required to break the contact between the surface and the finger. The Young’s modulus reflects the intrinsic property of the material. The arithmetic mean of the surface topography at each scale (SMa)) describes the surface topography of human fingers. The biophysics properties are measured in our previous research for the four age groups and for men and women^30^. The four age groups (G1, G2, G3, and G4) correspond to (26 ± 3, 35 ± 3, 45 ± 2, and 58 ± 6 years old), respectively, for men and women^30^. Two different touch gestures are analyzed (**A**) Left to right. (**B**) Top to bottom. Pearson’s statistical analyses: (+for r > 0.8, − for r < −0.8) highly correlated, (0) for no correlation, R indicates the correlation results. For both touch gestures, no linear correlation exists between MFCC and Fad. However, good correlations exist between MFCC and biophysical properties (Young’s modulus and topographic characterization SMa).

As the previous results show clear anisotropy of the mechanical properties of the human finger^30^, we have quantified a representative Young’s modulus for each touch gesture. For the left to right touch gesture, the representative Young’s modulus, *E*_*LR*_, (in the directions 90° and 270°, see Fig. 1A), ${E}_{LR}=({E}_{270^\circ }+{E}_{90^\circ })/2$^30^. In Fig. 6A, MFCCs are significantly and positively correlated to Young’s modulus (*E*_*LR*_) with age independently of the gender effect. The Pearson correlation coefficient shows values higher than 0.8, thus providing evidence of a linear correlation (MFCCs ∝ E). The results obtained on sample D are consistent with previous findings.

The Young’s modulus of the human finger and the roughness of its surface topography are predominant parameters of the vibratory signal obtained. The Young’s modulus of the finger, E, is significantly and positively correlated to age for men and women^30^. However, the maximum amplitude *SMa* decreased for women and increased for men with age^30^. The positive correlation between MFCCs with the finger’s mechanical and topographic properties for men can be explained by the fact that they increase with age. The results obtained for women indicated that the effect of Young’s modulus of the finger is higher than the surface topography on the vibratory signal obtained in the left to right touch gesture. This phenomenon can be explained by the large increment between the finger’s mechanical properties of the youngest and oldest age groups^30^ (≈60 kPa) (see Fig. 5). In this touch gesture, the Young’s modulus of the finger was identified as the most important biophysical property for women. The results obtained were consistent with the previous findings^49^. Indeed, no correlation could be found between the MFCCs and adhesion force (R < 0.8) (see Fig. 6A).

According to the results obtained on sample D in the left to right touch gesture, we conclude that Young’s modulus is the most important biophysical property for the vibratory signal obtained. Thus, Young’s modulus should be taken into consideration to understand age and gender effects in order to mimic the human finger.

#### Top to bottom touch gesture vs biophysical properties: Age and gender effects

For the top to bottom touch gesture, the representative Young’s modulus, *E*_*UD*_, (in 0° direction, see Fig. 1B) is ${E}_{TB}={E}_{0^\circ }$^30^. In Fig. 6B, MFCCs are significantly and negatively correlated with the finger’s Young’s modulus (*E*_*TB*_) with age in women. In addition, a positive correlation between MFCCs and the finger’s topographical properties can be seen in Fig. 6B. This phenomenon can be explained by the small difference between the mechanical properties of the youngest and oldest age groups^30^ (≈20 kPa) (see Fig. 5). In conclusion, fingerprint roughness was identified as the most important biophysical property for the top to bottom touch gesture for women. The positive correlation between MFCCs with mechanical and topographical properties for men can be explained by the increase in both of these biophysical properties of the finger with age. Indeed, no correlation could be found between the MFCCs and adhesion force (R < 0.8) (see Fig. 6B).

In conclusion, the interpretation of the results obtained on sample D is simple for men as Young’s modulus and *SMa* increase with age^30^, and they positively correlate well with the vibratory parameter (MFCCs). For women, Young’s modulus is positively correlated with age, and the *SMa* is inversely correlated with age^30^. Therefore, the evolution of vibratory parameters as a function of age allowed us to identify the biophysical property with the greatest influence in each touch gesture.

**Figure 7:**
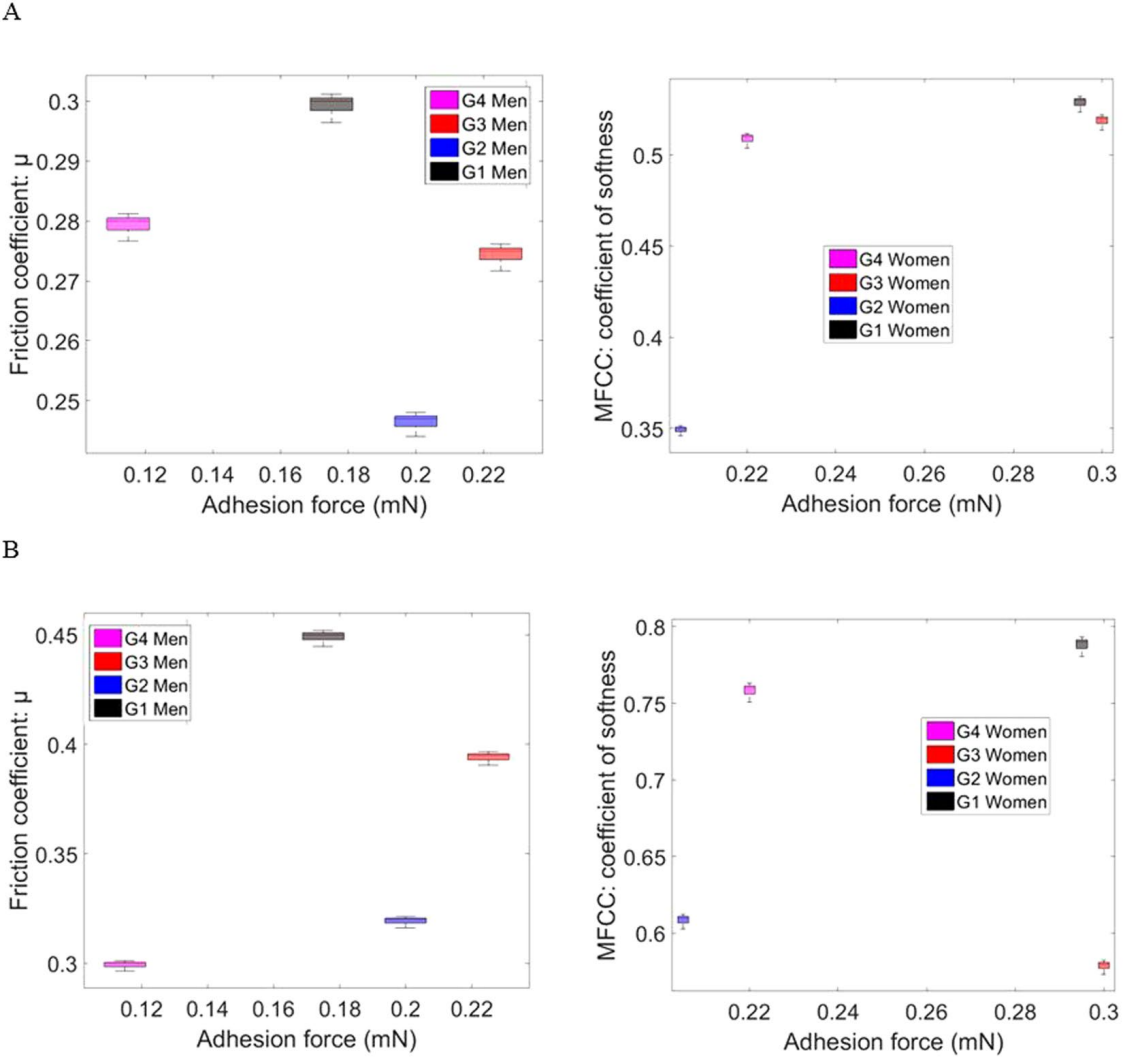
Correlation results between haptic touch parameter (µ) and adhesive force of the human finger for men and women and as a function of age are illustrated. The results are obtained with sample D. The adhesive force, Fad, is the force required to break the contact between the surface and the finger. This force is measured in our previous research for the four age groups and for men and women^30^. The four age groups (G1, G2, G3, and G4) correspond to (26 ± 3, 35 ± 3, 45 ± 2, and 58 ± 6 years old), respectively. Two different touch gestures are analyzed (**A**) Left to right. (**B**) Top to bottom. Pearson’s statistical analyses: (+for r > 0.8, - for r < −0.8) highly correlated, (0) for no correlation. For both touch gestures, no linear correlation exists between µ and Fad.

We studied the correlation between the friction coefficient and the three biophysical parameters, and we did not detect any correlation. This remark is in accordance to the literature, where there exists only a correlation between the friction coefficient and the adhesion force as we reported in the article. In the literature, the friction coefficient is correlated to the adhesive force in the case of friction between two materials. This correlation can be represented with the equation

$$\mu ={\mu }_{0}(1+\frac{{F}_{ad}}{{F}_{N}}),$$

where *μ*_0_ is the static coefficient of friction, *F*_*ad*_ is the adhesive force and *F*_*N*_ is the applied force^51–53^. Thus, we presented the correlation between the friction coefficient and adhesive force for both men and women and for different touch gestures (see Fig. 7). No correlation could be found between the friction coefficient and adhesion force (R < 0.8). The friction coefficient was affected by all finger biophysical properties; therefore, it is very difficult to use it to understand tactile perception as a function of age. Touch parameters can be described better with *L*_*a*_ and MFCCs than with µ.

### Touch gestures vs tactile perception: Age and gender influences

To understand tactile perception, vibratory signals and finger biophysical properties as a function of age and gender were investigated. In the literature, tactile perception declines with age for both men and women. On the other hand, the finger’s Young’s modulus increases with age independently of gender^30^. As the finger’s Young’s modulus increases, the ability of the finger’s skin to deform reduces so that E is inversely proportional to tactile perception (smaller contact area)^30^. Moreover, the results show a positive correlation between Young’s modulus and the vibratory parameters *L*_*a*_ and MFCCs. Therefore, we propose two main hypotheses, which we subsequently verify later:

1. The vibratory parameters (*L*_*a*_ and MFCCs) are inversely proportional to tactile perception.
2. Tactile perception anisotropy can be explained by the biophysical properties that have a greater effect on each touch gesture.

For men, the finger’s Young’s modulus was identified as the main parameter affecting the vibratory level and MFCCs parameter. As a function of age, the values of the vibratory parameters increased in both touch gestures. In Fig. 6, higher MFCCs can be observed in the top to bottom touch gesture, leading us to conclude that tactile perception is lower with this touch gesture. For women, our first hypothesis was always correct for the left to right touch gesture as the finger’s Young’s modulus was the predominant parameter in the results obtained (*L*_*a*_ and MFCCs). According to the literature, the tactile perception is higher for young women than for young men^54,55^. However, the results obtained for the vibratory parameters were higher for men, and according to our hypothesis, this finding means higher tactile perception for women. This finding is in accordance with the previous explanation about the effect of gender on tactile perception.

For the top to bottom touch gesture, the results demonstrated that fingerprint roughness was the most important factor affecting the vibratory parameters. The amplitude of fingerprint relief is positively proportional to tactile perception, as it generates more deformation and vibrations for the cutaneous receptors. In the literature, the gender effect has been demonstrated on *SMa* for the youngest age groups; the results also showed higher *SMa* values for women than for men^30^. This result is in line with the fact that young women have better tactile perception than young men^54,55^. For women, the finger’s Young’s modulus increases and the amplitude fingerprint roughness decreases with age, and both parameters are significantly and positively correlated to vibratory parameters^30^. However, tactile perception is significantly and negatively correlated with the finger’s Young’s modulus and positively correlated with *SMa*. Therefore, to understand tactile perception as a function of vibratory parameters, we should identify the main parameter affecting the vibratory signal.

**Figure 8:**
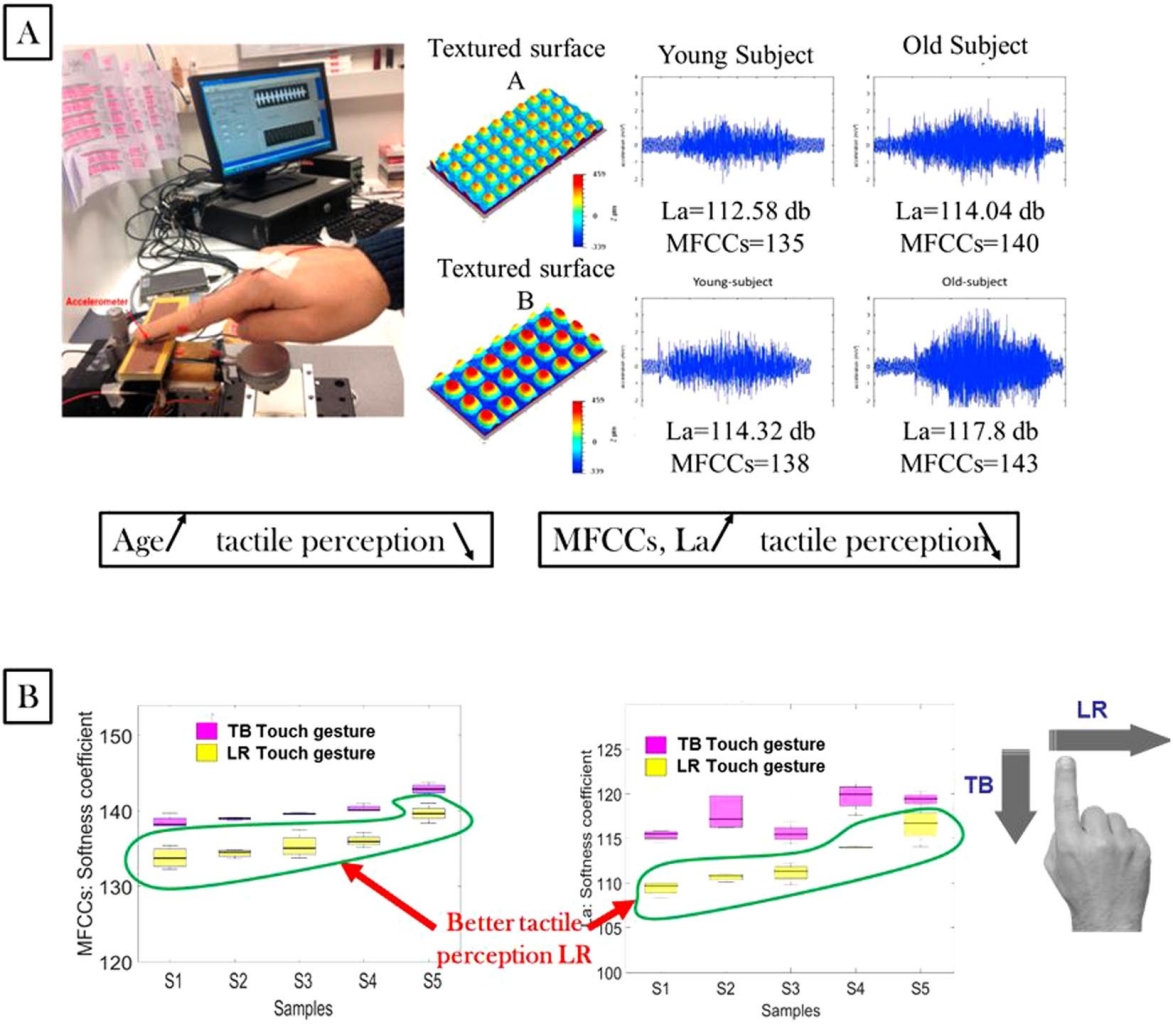
Touch gestures, tactile perception and vibratory parameter relationship (MFCCs). (**A**) The vibratory signal obtained for young and old subjects on two different surfaces with the tribohaptic system is shown. MFCC and La demonstrate the softness parameter, and it explains the relationship with the tactile perception. The tactile perception is inversely correlated to the MFCC. (**B**) The MFCC and La of five different plastic samples touched by G2 women (35 ± 3 years old) for two different touch gestures (left to right and top to bottom). The left to right touch gestures show better tactile perception with both La and MFCC.

Figure 8 confirms the previous hypothesis and conclusions in the previous section about the tactile perception and touch gestures relationship. In Fig. 8A, we demonstrated that the vibratory parameters (*L*_*a*_ and MFCCs) are positively correlated with age on two different surfaces. These results suggest that tactile perception is inversely proportional to vibratory parameters, which means higher tactile perception corresponds to lower MFCCs and *L*_*a*_ values. This conclusion is in accordance with the previous hypothesis and conclusions based on the comparison between the biophysical properties and vibratory parameters, where a better tactile perception was observed in the left to right touch gesture than in the top to bottom gesture (see Fig. 8B).

In conclusion, the left to right touch gesture provided better tactile perception than the top to bottom touch gesture according to our hypothesis and results. The panel classification in Table 1 proves our hypothesis as we observed the same classification of samples for all age groups as a function of their softness. In contrast, the results obtained with the top to bottom touch gesture varied as a function of age group. This can be explained by the tactile perception of the volunteers; it was better in the left to right touch gesture, which permitted assigning the right classification. These results are consistent with human nature^56^. Indeed, people use the left to right touch gesture with small roughness surfaces that need better perception and the top to bottom touch gesture with rough surfaces that do not require keen perception to be felt.

## Conclusion

The objective of this study was to understand the effects of age, gender and touch gestures on tactile perception via the biophysical properties of the human finger. This goal could not be achieved without the tribohaptic system used and the algorithms that objectify touch. The vibratory signal transmitted by the finger during the touch process was captured by a tribohaptic system and analyzed by different algorithms (*L*_*a*_, MFCCs and µ), one of which is new (MFCCs). Tissues and plastic samples were used to validate and prove the efficiency of the MFCC algorithm. The results of this algorithm allowed us to quantify the vibratory signal with a single value. This value is an image of the quality of the surface (softness). A correlation between the vibratory parameters and the biophysical properties of the finger allowed us to understand the impact of each on tactile perception. The results obtained showed the high performance of MFCCs for qualifying surface quality via a vibratory signal. In addition, it can be a good indicator of tactile perception, where the vibratory parameters (*L*_*a*_ and MFCCs) are inversely proportional to tactile perception.

Due to the anisotropy of the mechanical properties of the finger, two different touch gestures (left to right and top to bottom) were examined to understand the effect of touch direction on tactile perception. The touch gesture that allowed better tactile perception can be used in future developments to obtain better perception. The left to right touch presented the best tactile perception as it corresponds to lower MFCCs and *L*_*a*_. The different effects of the biophysical properties of the finger were identified for each touch gesture. In the left to right touch gesture, Young’s modulus was the parameter that had the greatest effect on tactile perception in men and women, meaning that Young’s modulus should be favored when considering age and gender effects in any development of an artificial tool to mimic the human finger.
